# Methyltransferase‐Like Protein 14 Attenuates Mitochondrial Antiviral Signaling Protein Expression to Negatively Regulate Antiviral Immunity via N^6^‐methyladenosine Modification

**DOI:** 10.1002/advs.202100606

**Published:** 2021-05-27

**Authors:** Fei Qin, Baoshan Cai, Jian Zhao, Lei Zhang, Yi Zheng, Bingyu Liu, Chengjiang Gao

**Affiliations:** ^1^ Key Laboratory of Infection and Immunity of Shandong Province and Department of Immunology School of Biomedical Sciences Shandong University Jinan Shandong 250012 P. R. China

**Keywords:** antiviral immunity, N6‐methyladenosine modification, methyltransferase‐like protein 14, mitochondrial antiviral signaling protein, mRNA stability

## Abstract

Mitochondrial antiviral signaling (MAVS) protein is the core signaling adaptor in the RNA signaling pathway. Thus, appropriate regulation of MAVS expression is essential for antiviral immunity against RNA virus infection. However, the regulation of MAVS expression at the mRNA level especially at the post transcriptional level is not well‐defined. Here, it is reported that the MAVS mRNA undergoes N^6^‐methyladenosine (m^6^A) modification through methyltransferase‐like protein 14 (METTL14), which leads to a fast turnover of MAVS mRNA. Knockdown or deficiency of METTL14 increases *MAVS* mRNA stability, and downstream phosphorylation of TBK1/IRF3 and interferon‐*β* production in response to RNA viruses. Compared to wild‐type mice, heterozygotes *Mettl14* ^+/−^ mice better tolerate RNA virus infection. The authors' findings unveil a novel mechanism to regulate the stability of MAVS transcripts post‐transcriptionally through m^6^A modification.

## Graphical abstract

This study reveals that METTL14 plays an important role in the retinoic acid‐inducible gene‐I‐like receptors (RLR)‐mediated innate antiviral response via m^6^A modification of *MAVS* mRNA. Deficiency of METTL14 increases MAVS mRNA stability and interferon‐*β* production in response to RNA viruses. m^6^A modification on *MAVS* transcripts by METTL14 negatively regulates the RLR‐induced innate immunity.

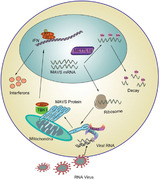

## 1 Introduction

The innate or non‐specific immune system is the forefront of host immune defense against pathogen invasion. During viral aggression, the innate immune system recognizes microorganisms via many pattern‐recognition receptors, which detect conserved microbial components including double‐stranded, known as pathogen‐associated molecular patterns, and ultimately initiates the induction of type I interferons (IFNs), pro‐inflammatory cytokines, and other downstream IFN‐stimulated genes (ISGs).^[^ ^1^ ^]^ Intracellular viral RNA is detected by the retinoic acid‐inducible gene‐I (RIG‐I)‐like receptors (RLRs), including retinoic‐acid‐inducible gene I (RIG‐I) and Melanoma differentiation‐associated gene 5 (MDA5). Studies have clarified that RIG‐I and MDA5 play pivotal roles in innate immune response to various types of RNA viruses, including human immunodeficiency virus (HIV), severe acute respiratory syndrome coronaviru (SARS), middle east respiratory syndrome coronavirus (MERS), Ebola virus, and SARS‐CoV‐2.^[^ ^2^, ^3^, ^4^, ^5^ ^]^ RIG‐I and MDA5 possess two caspase‐recruitment domains (CARDs) and a DExD/H‐box helicase domain. RIG‐I recruits a CARD‐containing adaptor mitochondrial antiviral signaling protein, mitochondrial antiviral signaling protein (MAVS, also known as IPS‐1, VISA or CARDIF), a mitochondrial integral outer‐membrane anchored protein with 540 amino acids.^[^ ^6^ ^]^ MAVS rapidly forms very large aggregates on the mitochondrial membrane upon viral infection, activating the cytosolic kinases IKK and serine/threonine‐protein kinase (TBK1), which consequently phosphorylate the transcription factors nuclear factor NF‐kappa‐B (NF‐*κ*B) and interferon regulatory factor 3 (IRF3), respectively. NF‐*κ*B and IRF3 transfer into the nucleus, where they cooperate to trigger the production of type I IFNs and other antiviral molecules.^[^ ^7^ ^]^

As a vital adaptor that propagates signals in the innate immune response, MAVS has been shown to be regulated by various post‐translational modifications, such as phosphorylation, ubiquitination, O‐GlcNAcylation, and succinylation.^[^ ^8^, ^9^, ^10^ ^]^ Upon modification, the space structure or protein quantity of MAVS could be influenced, following changes in interactions between MAVS and other molecules in RLR signaling. On the same time, control of its mRNA metabolism is also critical for managing the quantity of MAVS gene expression. For example, MiR‐3470b promotes bovine ephemeral fever virus replication via directly targeting MAVS, and miR‐27a inhibits MAVS expression, promoting the replication of vesicular stomatitis virus (VSV).^[^ ^11^, ^12^ ^]^ Recent studies demonstrated that post‐transcriptional regulation of mRNA, such as m^6^A or 5‐methylcytosine (m^5^C) modifications, help cells respond more rapidly to external signaling at the transcriptional level.^[^ ^13^, ^14^, ^15^ ^]^ However, the molecular regulatory mechanism of post‐transcriptional modification on *MAVS* mRNA remains indistinct.

Methylation of adenosine at the N^6^ position (m^6^A) of RNA has been identified as the most common mammalian mRNA modification, which can modulate enormous genes expression and regulate extensive biological activities including metabolism, tumor progression, circadian clock, and DNA damage response.^[^ ^16^, ^17^ ^]^ The m^6^A modification is a dynamic and reversible change that can be controlled by proteins acting as “writers,” “erasers,” and “readers.” m^6^A is installed by m^6^A methyltransferases (writers: methyltransferase‐like protein 3‐METTL3, METTL14, Pre‐mRNA‐splicing regulator‐WTAP and others) and eliminated by m^6^A demethylases (erasers: fat mass and obesity‐associated protein‐FTO and alkylated DNA repair protein alkB homolog 5‐ALKBH5).^[^ ^18^ ^]^ Reader proteins recognize m^6^A and participate in the degradation of downstream RNA and translation.^[^ ^19^ ^]^ The roles of m^6^A modification include regulating mRNA stability, splicing, transport, localization, and translation, as well as RNA‐protein interactions.^[^ ^16^, ^17^, ^18^, ^19^ ^]^

Several studies have reported that m^6^A modifications play an important role in innate antiviral immunity. It has been reported that m^6^A modification served as an inactive regulator of type I IFN response by directly guiding the fast turnover of interferon alpha (IFNΑ) and interferon beta (IFNB) mRNA.^[^ ^20^ ^]^ But, whether other molecules in innate antiviral signaling pathway can be regulated by m^6^A modification is not clear. Therefore, it is of great scientific significance to explore the mechanism of m^6^A modification on key adaptors in the RLRs signaling pathway.

In this study, using siRNA knockdown and *Mettl14* deficient mice, we demonstrated that *Mettl14*‐mediated m^6^A modification of *Mavs* mRNA could directly regulate *Mavs* mRNA stability, therefore, phosphorylation of TBK1/IRF3 and IFN‐*β* production in response to RNA viruses were directly regulated. Importantly, compared to wild‐type mice, heterozygotes *Mettl14* ^+/−^ mice better tolerate RNA virus infection. Our findings suggested that m^6^A modification on MAVS transcripts by METTL14 negatively regulates RLR‐induced innate immunity.

## 2 Results

### 2.1 Methyltransferase‐Like Protein 14‐Mediated Modification Inhibits RLR‐Induced Innate Immunity Signaling

To investigate whether other molecules upstream of type I IFN were affected by m^6^A, we first measured the TBK1 and IRF3 phosphorylation after RNA virus infection, which are upstream molecules essential for the expression of type I IFNs.

We transfected siMETTL14 into primary peritoneal macrophages to knock down the expression of METTL14. Consistently, we found that global m^6^A level in peritoneal macrophages was substantially decreased in the presence of siMETTL14 compared to control siRNA (siCtrl) as measured by m^6^A dot blot assays (Figure S1A, Supporting Information). As reported, we found the level of *Ifnb1* mRNA (encoding IFN‐*β*) was significantly elevated upon infection with Sendai virus (SeV) or simulation with 5′‐pppRNA in siMETTL14 transfected peritoneal macrophages compared to that in control siRNA (siCtrl) transfected cells (Figure S1B, Supporting Information). Interestingly, we found siRNA knockdown of METTL14 expression in primary peritoneal macrophages also increased the phosphorylation of IRF3 and TBK1 upon stimulation with 5′‐pppRNA (Figure S1C, Supporting Information).

**Figure 1:**
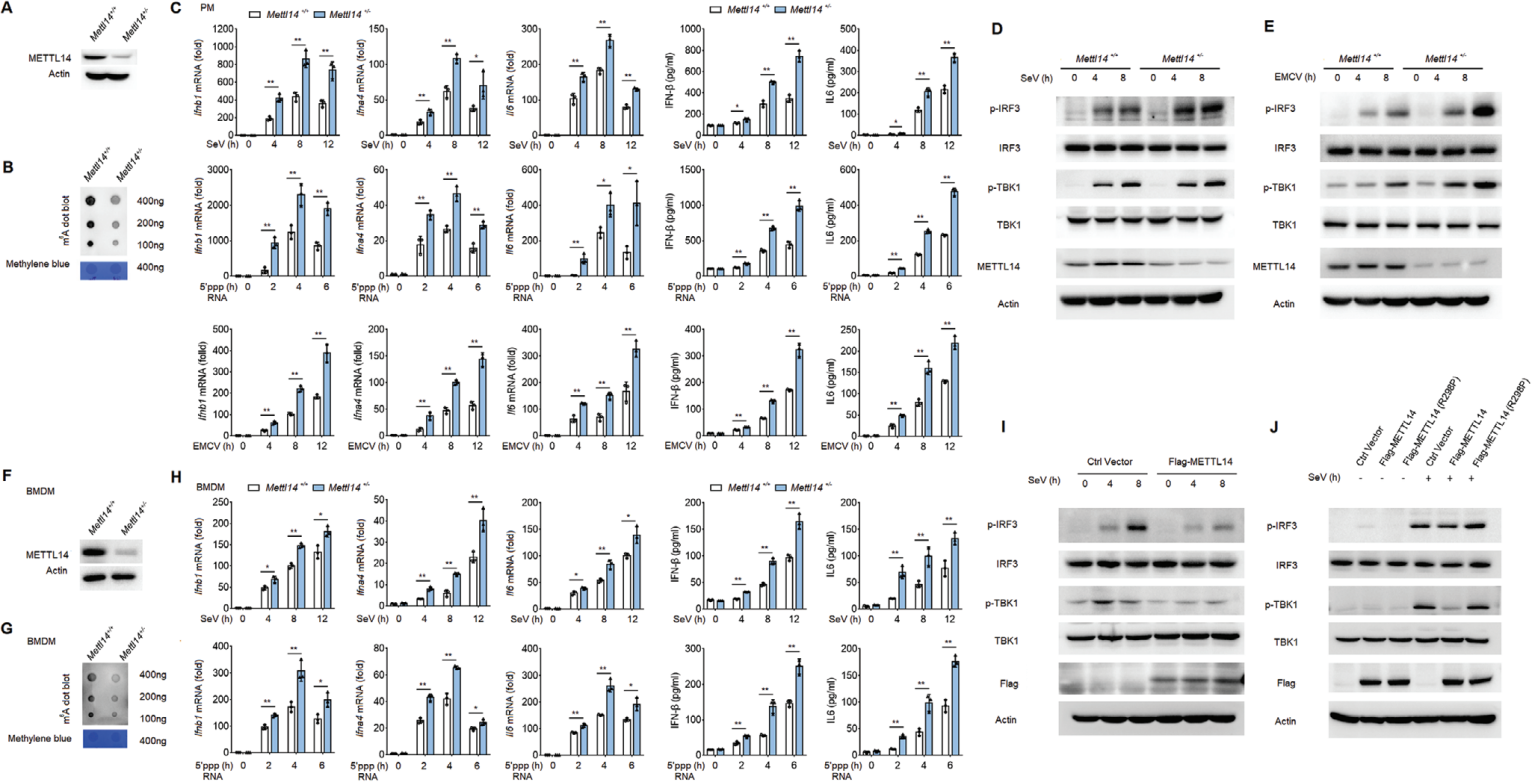
METTL14‐mediated m^6^A modification inhibits RLR‐induced innate immunity signaling. A) Immunoblot analysis of METTL14 in *Mettl14* ^+/+^ and *Mettl14* ^+/−^ peritoneal macrophages. B) m^6^A dot blot assays of *Mettl14* ^+/+^ and *Mettl14* ^+/−^ peritoneal macrophages, methylene blue staining (as loading control). C) qPCR analysis of *Ifnb1*, *Il6*, or *Ifna4* mRNA in *Mettl14* ^+/+^ and *Mettl14* ^+/−^ peritoneal macrophages, followed by infection with SeV or EMCV or stimulation with 5′‐ppp RNA for the indicated times. D) Immunoblot analysis of phosphorylated and total IRF3 and TBK1 in lysates of *Mettl14* ^+/+^ and *Mettl14* ^+/−^ peritoneal macrophages infected with SeV for the indicated times. E) Immunoblot analysis of phosphorylated and total IRF3 and TBK1 in lysates of *Mettl14* ^+/+^ and *Mettl14* ^+/−^ peritoneal macrophages infected with EMCV for the indicated times. F) Immunoblot analysis of METTL14 in *Mettl14* ^+/+^ and *Mettl14* ^+/−^ BMDMs. G) m^6^A dot blot assays of *Mettl14* ^+/+^ and *Mettl14* ^+/−^ BMDMs, methylene blue staining (as loading control). H) qPCR analysis of *Ifnb1*, *Il6*, or *Ifna4* mRNA in *Mettl14* ^+/+^ and *Mettl14* ^+/−^ BMDMs, followed by infection with SeV or stimulation with 5′‐pppRNA for the indicated times. I) Immunoblot analysis of phosphorylated and total IRF3 and TBK1 in lysates of HEK293T cells transfected with control plasmid or plasmid expressing Flag‐METTL14 (above blots) for 24 h, followed by infection with SeV for the indicated times. J) Immunoblot analysis of phosphorylated and total IRF3 and TBK1 in lysates of HEK293T cells transfected with control plasmid or plasmid expressing Flag‐METTL14 or Flag‐METTL14 (R298P) (above blots), followed by SeV infection for 8 h. Data information: Data are presented as mean ± S.D. Two‐tailed unpaired Student's *t*‐test; *P < 0.05; **P < 0.01 (C,H).

We generated *Mettl14*‐deficient mice using CRISPR/Cas9 technology. Unfortunately, we could not obtain homozygous *Mettl14* ^−/−^ mice through mating between heterozygous *Mettl14* ^+/−^ mice, which is consistent with previous studies showing that depletion of *Mettl14* resulted in embryonic lethality early in gestation.^[^ ^21^ ^]^ However, the heterozygous *Mettl14* ^+/−^ mice are viable. Western blotting and m^6^A dot analysis showed that the protein expression of METTL14 and the global m^6^A level were substantially decreased in macrophages prepared from *Mettl14* ^+/−^ mice, compared to that from *Mettl14* ^+/+^ mice (**Figure** **1**A,B). Thus, we employed *Mettl14* ^+/+^ and *Mettl14* ^+/−^ mice in the following experiments. Similar to the data in siRNA knockdown cells, we observed enhanced expression of *Ifnb1*, *Il6*, and *Ifna4* mRNA and production of IFN‐*β* and IL6 protein in peritoneal macrophages prepared from *Mettl14* ^+/−^ mice upon infection with SeV, Encephalomyocarditis virus (EMCV), or simulation with 5′‐pppRNA, compared to that from wild‐type littermates (Figure 1C). Again, we found that phosphorylation of TBK1 and IRF3 induced by SeV infection or 5′‐pppRNA stimulation was remarkably increased in *Mettl14* ^+/−^ primary peritoneal macrophages, compared to that in wild‐type macrophages (Figure 1D and Figure S1C, Supporting Information). And beyond that, the phosphorylation of TBK1 and IRF3 induced by EMCV infection was upregulated in *Mettl14* deficient primary peritoneal macrophages (Figure 1E). However, METTL14 deficiency did not affect the phosphorylation of TBK1 and IRF3 induced by herpes simplex virus 1 (HSV‐1; Figure S1E, Supporting Information).

Similarly, we found the protein expression of METTL14 and the global m^6^A level were substantially decreased in bone‐marrow‐derived macrophages (BMDMs) prepared from *Mettl14* ^+/−^ mice, compared to that from *Mettl14* ^+/+^ mice (Figure 1F,G). The expression of *Ifnb1*, *Il6*, and *Ifna4* were increased in BMDMs from *Mettl14* ^+/−^ mice after infection with SeV or treatment with 5′‐pppRNA, compared to that in wild‐type BMDMs from *Mettl14* ^+/+^ littermates (Figure 1H). We also observed that *Mettl14* ^+/−^ BMDMs showed higher phosphorylation levels of IRF3 and TBK1 upon SeV infection, relative to *Mettl14* ^+/+^ BMDMs (Figure S1F, Supporting Information).

Further, we showed that SeV‐induced phosphorylation of IRF3 and TBK1 was attenuated by Flag‐METTL14 overexpression in HEK293T cells (Figure 1I). Notably, cells expressing Flag‐METTL14 mutant R298P, which has been shown to have lost the ability for METTL14 catalytic activity and mRNA substrate recognition of the methyltransferase complex,^[^ ^21^, ^22^ ^]^ could not inhibit SeV‐induced phosphorylation of IRF3 and TBK1 (Figure 1J). These data suggested that METTL14 might regulate the m^6^A methylation of upstream adaptors in the RLR signaling pathway, and then regulate the production of multiple cytokines including IFNs and interleukins.

### 2.2 Methyltransferase‐Like Protein 14 Attenuates Mitochondrial Antiviral Signaling Protein Expression

**Figure 2:**
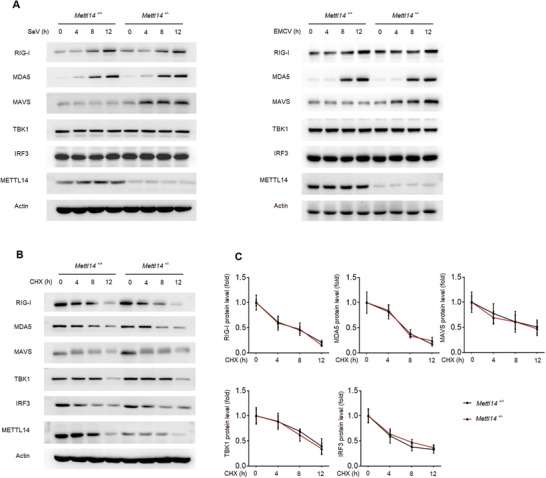
METTL14 attenuates MAVS protein expression. A) Immunoblot analysis of the main adaptors in RLRs signaling pathway in *Mettl14* ^+/+^ and *Mettl14* ^+/−^ peritoneal macrophages infected with SeV or EMCV for the indicated times. B,C) Immunoblot analysis (B) and quantification (C) of innate main adaptors degradation kinetics in *Mettl14* ^+/+^ and *Mettl14* ^+/−^ peritoneal macrophages treated with the protein synthesis inhibitor CHX for the indicated times.

To identify molecules in the RLR signaling pathway regulated through METTL14‐mediated m^6^A modification, we assessed the expression of the key molecules in the RLRs signaling pathway. We prepared primary peritoneal macrophages from *Mettl14* ^+/+^ and *Mettl14* ^+/−^ mice followed with SeV infection for different times. Western blotting analysis showed that SeV infection in WT macrophages increased the protein level of RIG‐I and MDA5 (**Figure** **2**A), whose increased expression after virus infection has been reported previously.^[^ ^23^ ^]^ While, the protein level of MAVS, TBK1, and IRF3 is not greatly changed upon virus infection in WT macrophages (Figure 2A). However, compared to wide type macrophages, knockout of *Mettl14* significantly increased the MAVS protein expression in macrophages after SeV or EMCV virus infection (Figure 2A). The protein level of RIG‐I and MDA5 was similarly increased in *Mettl14* ^+/−^ macrophages as that in *Mettl14* ^+/+^ macrophages (Figure 2A). Knockout of *Mettl14* had no effect on the expression of TBK1 and IRF3 proteins (Figure 2A). siRNA knockdown of METTL14 expression in primary peritoneal macrophages also elevated MAVS protein level after virus infection (Figure S2A, Supporting Information). Further, we showed that knockout of *Mettl14* expression in BMDMs also increased MAVS protein level, while, the protein level of RIG‐I, MDA5, TBK1 and IRF3 was not affected (Figure S2B, Supporting Information). These data suggested that METTL14 specifically regulates MAVS protein expression, which is an upstream molecule of TBK1 and IRF3 in the RLR‐induced innate signaling pathway.

To further investigate how METTL14 regulates MAVS protein expression, we first measured the protein degradation rate of RIG‐I, MAVS, TBK1, and IRF3. *Mettl14* ^+/+^ and *Mettl14* ^+/−^ peritoneal macrophages were first stimulated by SeV infection for 8 h, followed by treatment with cycloheximide (CHX) to inhibit protein expression for the indicated times. In these assays, we found the degradation rate of RIG‐I, MAVS, TBK1, and IRF3 was not affected by *Mettl14* deficiency, indicating that METTL14 could not regulate MAVS protein stability to increase MAVS protein level in *Mettl14* deficient macrophages (Figure 2B,C).

### 2.3 Methyltransferase‐Like Protein 14 Promotes Mitochondrial Antiviral Signaling Protein (*MAVS)* mRNA Decay

**Figure 3:**
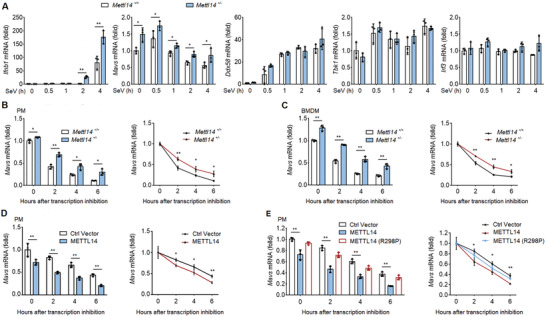
METTL14 promotes *MAVS* mRNA decay. A) qPCR analysis of *Ifnb1*, *Mavs*, *Ddx58*, *Tbk1*, and *Irf3* mRNAs in *Mettl14* ^+/+^ and *Mettl14* ^+/−^ peritoneal macrophages, followed by infection with SeV for the indicated times. B) qPCR analysis of *Mavs* mRNAs (left) and *Mavs* mRNA degradation (right) in *Mettl14* ^+/+^ and *Mettl14* ^+/−^ peritoneal macrophages infected with SeV for 8 h, followed by treatment with actinomycin‐D for the indicated times. C) qPCR analysis of *Mavs* mRNAs (left) and *Mavs* mRNA degradation (right) in *Mettl14* ^+/+^ and *Mettl14* ^+/−^ BMDMs infected with SeV for 8 h, followed by treatment with actinomycin‐D for the indicated times. D) qPCR analysis about the degradation of *Mavs* mRNA in macrophages reconstituted with empty vector and METTL14 using lentivirus followed by infection with SeV for 8 h and treatment with actinomycin‐D as indicated times. E) qPCR analysis about the degradation of *Mavs* mRNA in macrophages reconstituted with empty vector, METTL14, and mutant METTL14 (R298P) using lentivirus followed by infection with SeV for 8 h and treatment with actinomycin‐D as indicated times. Data information: Data are presented as mean ± S.D. Two‐tailed unpaired Student's *t*‐test; *P < 0.05; **P < 0.01 (A–E).

We then measured the steady state mRNA levels of *Ddx58*, *Mavs*, *Tbk1*, and *Irf3* in *Mettl14* ^+/+^ and *Mettl14* ^+/−^ macrophages before and after SeV infection. We found SeV infection increased the steady state mRNA level of *Ddx58* in both *Mettl14* ^+/+^ and *Mettl14* ^+/−^ macrophages in a similar kinetics after virus infection (**Figure** **3**A). The steady state mRNA levels of *Tbk1* and *Irf3* in both *Mettl14* ^+/+^ and *Mettl14* ^+/−^ macrophages were not changed after virus infection (Figure 3A). The steady state mRNA level of *Mavs* gradually decreased upon virus infection in WT macrophages (Figure 3A). However, the *Mavs* mRNA level was substantially higher in *Mettl14* ^+/−^ macrophages before and after SeV infection, indicating *Mettl14* regulates *Mavs* mRNA expression (Figure 3A). As a control, we found SeV infection‐induced *Ifnb1* mRNA was further elevated in *Mettl14* deficient macrophages (Figure 3A), consistent with the report that *Ifnb1* mRNA is an m^6^A target.^[^ ^20^ ^]^

Modification of mRNA by m^6^A has been reported to regulate mRNA decay.^[^ ^24^ ^]^ Thus, we measured the *Mavs* mRNA stability in *Mettl14* ^+/+^ and *Mettl14* ^+/−^ macrophages. *Mettl14* ^+/+^ and *Mettl14* ^+/−^ peritoneal macrophages were first stimulated by SeV infection for 8 h, followed by treatment with actinomycin‐D for the indicated times. Quantitative reverse transcription PCR (qPCR) analysis of *Mavs* mRNA showed that both *Mavs* mRNA stability and steady mRNA level were increased in *Mettl14* ^+/−^ macrophages, relative to levels of *Mavs* mRNA in *Mettl14* ^+/+^ macrophages (Figure 3B). siRNA knockdown of METTL14 expression in macrophages also increased the stability of *Mavs* mRNA (Figure S3A, Supporting Information). We further observed that the stability and expression of *Mavs* mRNA were significantly increased in *Mettl14* ^+/−^ BMDMs compared to that in *Mettl14* ^+/+^ BMDMs (Figure 3C). siRNA knockdown of METTL14 in THP‐1 cells also delayed *MAVS* mRNA degradation and increased its accumulation in THP‐1 cells (Figure S3B, Supporting Information). On the contrary, the stability and expression of *Mavs* mRNA were reduced in METTL14 ectopically forced expressed macrophages (Figure 3D). Similarly, the stability and expression of *MAVS* mRNA were decreased in HEK293T cells with the overexpression of wild‐type Flag‐tagged METTL14 (Figure S3E, Supporting Information). Notably, compared to wild‐type METTL14, the METTL14‐R298P mutant lost the ability to promote the degradation of *MAVS* mRNA (Figure 3E and Figure S3E, Supporting Information). To further confirm that METTL14 regulates *MAVS* at the post‐transcriptional level, we also measured the initiation of MAVS transcription. We extracted the nascent RNA from the cell nucleus infected with SeV and labeled with 5‐ethyluridine (EU) metabolic pulse for 30 min. We found that the newly synthesized RNA production of *Ddx58*, *Mavs*, *Tbk1*, *Ifnb1*, *and Irf3* was not influenced by *Mettl14* deficiency, indicating *Mettl14* could not upregulate *Mavs* mRNA transcription to increase MAVS mRNA level in *Mettl14* ^+/−^ macrophages as that in *Mettl14* ^+/+^ macrophages (Figure S3F, Supporting Information).

Taken together, these data demonstrated that METTL14, as a functionally active methyltransferase, decreases *MAVS* mRNA stability and its accumulation thereby reducing MAVS protein expression.

### 2.4 Methyltransferase‐Like Protein 14 Catalyzes m^6^A Modification of Mitochondrial Antiviral Signaling Protein (*Mavs)* mRNA

**Figure 4:**
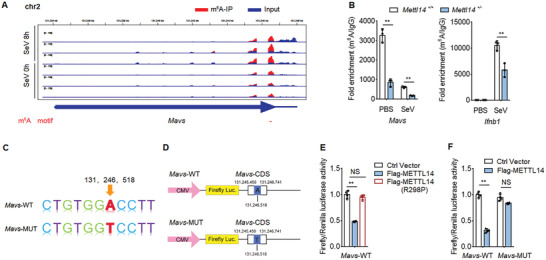
METTL14 catalyzes m^6^A modification of *Mavs* mRNA. A) RNA‐seq of *Mavs* mRNA in input RNA and m^6^A immunoprecipitated RNA from peritoneal macrophages infected with SeV for 8 h; m^6^A motif sequences that correspond to an immunoprecipitate‐enriched region are marked in red. B) The enrichment of *MAVS* mRNA in *Mettl14* ^+/+^ and *Mettl14* ^+/−^ macrophages with anti‐m^6^A antibody, followed by m^6^A‐RIP‐qPCR analysis, with IgG as a negative control. C) Schematic plot of the *Mavs*‐WT or *Mavs*‐MUT mRNA nucleotide sequence. D) Schematic plot of the luciferase reporter of *Mavs*‐WT or *Mavs*‐MUT. E) Relative luciferase activity of *Mavs*‐WT was measured after cotransfection with Flag‐METTL14, Flag‐METTL14‐R298P, or control vector in HEK293T cells. Cell lysis was quantified for firefly luciferase activity, with the normalization to Renilla luciferase activity as an inner control. F) Relative luciferase activity of *Mavs*‐WT and a mutant vector carrying mutation in the m^6^A site were measured after cotransfection with Flag‐METTL14 or control vector in HEK293T cells. Cell lysis was quantified for firefly luciferase activity, with the normalization to Renilla luciferase activity as an internal control. Data information: Data are presented as mean ± S.D. Two‐tailed unpaired Student's *t*‐test; *P < 0.05; **P < 0.01; NS: no significance (B,E,F).

The above finding that METTL14 regulates the stability of *MAVS* mRNA prompted us to investigate whether *MAVS* mRNA was m^6^A‐modified by METTL14. We first performed methylated RNA immunoprecipitation (RIP) sequencing (MeRIP‐seq) to profile the transcriptome‐wide m^6^A modification sites in wild‐type macrophages before and after SeV infection. The data displayed that *Ifnb1*, *Tarf3*, and *Traf6* mRNA were modified by m^6^A as previously reported (Figure S4, Supporting Information),^[^ ^20^, ^25^ ^]^ which confirmed the reliability of these assays. Interestingly, this transcriptome‐wide m^6^A‐seq assay showed that the m^6^A modification is indeed present in the *Mavs* mRNA before and after SeV infection, and we found a putative m^6^A modification site, which was adjacent to the stop codon (**Figure** **4**A). In contrast, this analysis revealed no m^6^A modification on *Ddx58 (Rig‐I)*, *Ifih1 (Mda5)*, and *Tbk1* mRNA transcripts (Figure S4, Supporting Information). If the m^6^A modification changes by SeV infection were mediated by METTL14, it needs a further verification.

We next utilized RIP followed by qPCR (RIP‐qPCR) to verify the transcriptome‐wide m^6^A‐seq data. We observed that *Mavs* mRNA was m^6^A‐modified in macrophages before SeV infection, and SeV infection decreased m^6^A modification on *Mavs* mRNA. Importantly, m^6^A‐modified *Mavs* mRNA levels were substantially weakened in the absence of *Mettl14* (Figure 4B, left). Again, we found m^6^A modification was present in *Ifnb1* mRNA especially after SeV infection, and the increase of m^6^A modification decreased in *Mettl14* deficient macrophages (Figure 4B, right). To evaluate the m^6^A modifications site on *Mavs* mRNA by METTL14 and if this modification directly regulates *Mavs* mRNA stability, we designed a series of experiments. Combining the transcriptome‐wide m^6^A‐seq data and a software prediction, we found the putative m^6^A modification site (131246518 in Chromosome 2), which was adjacent to the stop codon (Figure 4C). We then generated a luciferase reporter that included the wild‐type *Mavs* CDS truncation sequence (nucleic acid from 131245450 to 131246741 in Chromosome 2, named *Mavs*‐WT) or a mutant luciferase reporter CDS truncation sequence (named *Mavs*‐MUT), in which we replaced the adenine into thymine at the 131246518 site of *Mavs* (Figure 4D). Luciferase reporter assays showed that expressing wild‐type Flag‐METTL14 markedly weakened *Mavs*‐WT luciferase activity, which was not observed with mutated METTL14‐R298P (Figure 4E). Importantly, ectopical expression of Flag‐METTL14 dampened luciferase activity of reporter constructs with wild‐type *Mavs*, whereas the restraint abolished when the m^6^A site mutation (*Mavs*‐MUT) was made (Figure 4F). Collectively, these findings prove that the regulation of MAVS stability and innate immunity signaling mediated by METTL14 depends on the methyltransferase activation and putative m^6^A site on the *Mavs* mRNA.

### 2.5 Methyltransferase‐Like Protein 14 Inhibits Cellular Antiviral Response to RNA Virus

RLRs‐mediated IFN‐*β* production plays essential roles in the innate immune responses against RNA viral infection. To investigate the function of METTL14 on antiviral responses in vivo, VSV was used as an RNA virus. Similar to SeV infection, VSV infection induced elevated level of *Ifnb1* mRNA expression in primary peritoneal macrophages transfected with siMETTL14, compared to that in macrophages transfected with control siRNA (siCtrl) (Figure S5A, Supporting Information). Accordingly, VSV mRNA measured by qPCR and VSV titers measured by plaque assay substantially decreased in siMETTL14 transfected peritoneal macrophages compared to siCtrl transfected cells (Figure S5A, Supporting Information). Vesicular stomatitis virus G protein (VSV‐G) also decreased in siMETTL14 transfected macrophages compared to that in the control siRNA transfected cells (Figure S5B, Supporting Information). Similarly, knockdown of METTL14 expression using siRNA in THP‐1 cells increased *IFNB1* expression and decreased VSV replication after VSV infection (Figure S5C, Supporting Information). VSV‐G also decreased in METTL14‐knockdown THP‐1 cells (Figure S5D, Supporting Information).

**Figure 5:**
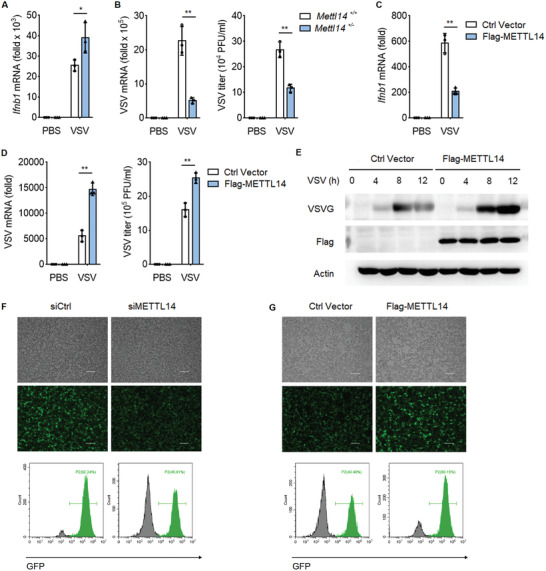
METTL14 inhibits cellular antiviral response to RNA virus. A) qPCR analysis of *Ifnb1* mRNA in *Mettl14* ^+/+^ and *Mettl14* ^+/−^ peritoneal macrophages infected with VSV (MOI, 0.1) for 12 h. B) qPCR analysis of VSV mRNA (left) and plaque assay of VSV titers (right) in *Mettl14* ^+/+^ and *Mettl14* ^+/−^ peritoneal macrophages infected with VSV (MOI, 0.1) for 12 h. C) qPCR analysis of *Ifnb1* mRNA in HEK293T cells transfected with indicated plasmids for 24 h, followed by infection with VSV (MOI, 0.1) for 12 h. D) qPCR analysis of VSV mRNA (left) and plaque assay of VSV titers (right) in HEK293T cells transfected as in (C), followed by infection with VSV (MOI, 0.1) for 12 h. E) Immunoblot analysis of VSV glycoprotein (VSV‐G) in HEK293T cells transfected as in (C), followed by infection with VSV for the indicated times. F) Fluorescence microscopy (above) and flow cytometry analysis (bottom) of VSV‐GFP replication in HEK293T cells transfected with control siRNA (siCtrl) or siRNA targeting METTL14 (siMETTL14) for 48 h, followed by infection with VSV‐GFP (MOI, 0.1) for 12 h (bright‐field, upper; fluorescence, bottom). Scale bars, 100 µm. G) Fluorescence microscopy (above) and flow cytometry analysis (bottom) of VSV‐GFP replication in HEK293T cells transfected as in (C), followed by infection with VSV‐GFP (MOI, 0.05) for 12 h (bright‐field, upper; fluorescence, bottom). Scale bars, 100 µm. Data information: Data are presented as mean ± S.D. Two‐tailed unpaired Student's *t*‐test; *P < 0.05; **P < 0.01 (A–D).

We also prepared primary peritoneal macrophages from *Mettl14* ^+/+^ mice and *Mettl14* ^+/−^ mice followed infection with VSV. We found *Ifnb1* mRNA expression was upregulated in peritoneal macrophages prepared from *Mettl14* ^+/−^ mice relative to that from *Mettl14* ^+/+^ mice after VSV infection (**Figure** **5**A). Accordingly, VSV replication was potently inhibited in *Mettl14* ^+/−^ peritoneal macrophages (Figure 5B).

To further investigate the role of METTL14 on virus replications, we transfected METTL14 expression plasmid into HEK293T cells and then the cells were infected with VSV. Overexpression of Flag‐METTL14 decreased *IFNB1* mRNA level upon infection with VSV (Figure 5C). Accordingly, the replication of VSV as measured by VSV‐specific mRNA, VSV titers, and VSV‐G, was substantially increased in METTL14 transfected cells (Figure 5D,E). We further used VSV‐GFP virus to infect HEK293T cells and measured the function of METTL14 on VSV‐GFP infection. Fluorescence microscopy showed that siRNA knockdown of METTL14 expression in HEK293T cells inhibited VSV‐GFP levels relative to control siRNA transfected cells (Figure 5F). In contrast, transfection of METTL14 expression plasmid increased replication of VSV‐GFP in HEK293T cells (Figure 5G). All together, these data suggested that METTL14 negatively regulates RLRs‐mediated IFN‐*β* production and innate antiviral immune responses.

### 2.6 Methyltransferase‐Like Protein 14 Negatively Regulates Antiviral Response to RNA Virus In Vivo

**Figure 6:**
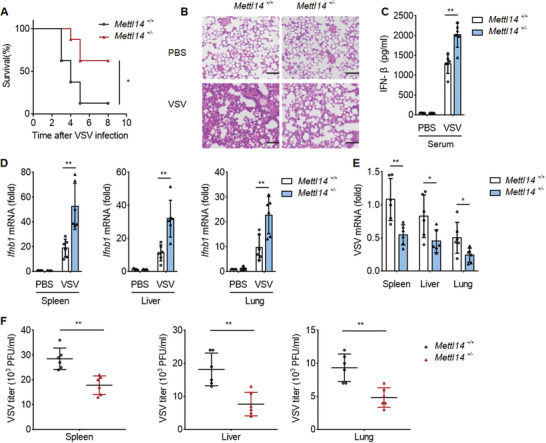
METTL14 negatively regulates antiviral response to RNA virus in vivo. A) Survival of *Mettl14* ^+/+^ and *Mettl14* ^+/−^ mice infected intravenously with VSV (1 × 10^8^ PFU per mouse). B) Hematoxylin‐and‐eosin‐stained images of lung sections of *Mettl14* ^+/+^ and *Mettl14* ^+/−^ mice infected by intraperitoneal injection of VSV (1 × 10^8^ PFU per mouse) for 48 h, Scale bars, 100 µm. C) ELISA analysis of IFN‐*β* of Serum from *Mettl14* ^+/+^ and *Mettl14* ^+/−^ mice infected by intraperitoneal injection of VSV (1 × 10^8^ PFU per mouse) for 8 h. D) qPCR analysis of *Ifnb1* mRNA (middle right) of the spleen (left), liver (middle), and lungs (right) from *Mettl14* ^+/+^ and *Mettl14* ^+/−^ (six per group) mice infected by intraperitoneal injection of VSV (1 × 10^8^ PFU per mouse) for 48 h. E) qPCR analysis of VSV RNA of the spleen (left), liver (middle), and lungs (right) from *Mettl14* ^+/+^ and *Mettl14* ^+/−^ mice infected by intraperitoneal injection of VSV (1 × 10^8^ PFU per mouse) for 48 h. F) Plaque assay of VSV titers of the spleen, liver, and lungs from *Mettl14* ^+/+^ and *Mettl14* ^+/−^ mice infected by intraperitoneal injection of VSV (1 × 10^8^ PFU per mouse) for 48 h. Each symbol represents an individual mouse; small horizontal lines indicate the mean. Data information: Data are presented as mean ± S.D. Log‐rank test (A); Two‐tailed unpaired Student's *t*‐test; *P < 0.05; **P < 0.01 (C–F).

To investigate the role of METTL14 on antiviral immunity in physiological condition, we challenged *Mettl14* ^+/+^ and *Mettl14* ^+/−^ mice with VSV. *Mettl14* ^+/−^ mice showed improved survival than their WT littermates during viral infection (**Figure** **6**A). Hematoxylin and‐eosin staining showed less damage in the lungs of *Mettl14* ^+/−^ mice compared to that of *Mettl14* ^+/+^ mice (Figure 6B). ELISA assays showed increased IFN‐*β* production in the sera from *Mettl14* ^+/−^ mice compared to that from WT mice (Figure 6C). qPCR analysis also showed that *Mettl14* ^+/−^ mice had substantially increased *Ifnb1* mRNA in the lung, liver, and spleen (Figure 6D). Accordingly, the replication of VSV in the lung, liver, and spleen was attenuated as indicated by VSV mRNA measured by qPCR and viral titers measured by plaque assays (Figure 6E,F). In order to validate the role of macrophages in our viral infection model, clodronate liposomes were treated for 3 days to delete macrophages before viral infection. Flow cytometry analysis showed the successful deletion of macrophages in the blood and spleen (Figure S6A, Supporting Information). In the absence of macrophages, *Mettl14* ^+/+^ and *Mettl14* ^+/−^ mice showed the same antiviral ability to VSV challenge, which was evaluated based on survival rate, IFN‐*β* secretion in serum, and viral burden in lung, liver, and spleen (Figure S6B–D, Supporting Information). This indicates that macrophages are the key to the antiviral response against viral infections in METL14‐deficient mice. Taken together, these data demonstrated that *Mettl14* facilitates VSV replication in vivo.

## 3 Discussion

Type I IFN production is the hallmark of immunity against a variety of viral infections, and it results in the production of autocrine and paracrine antiviral factors. Type I IFN response can be regulated by various enhancement and inhibitory signals that induce robust and powerful antiviral responses. The effect of m^6^A modification in the innate antivirus signaling pathway is not fully understood.

One study showed that attenuating m^6^A writer METTL3 and reader YTHDF2 could directly reduce the m^6^A modification of mouse *Ifnb1* mRNA, thereby accelerating its mRNA degradation, which decreased ISGs expression and weakened antiviral effect during viral infection.^[^ ^20^ ^]^ It was also reported that knockdown of METTL14 using siRNA increased the production and stability of nascent *Ifnb1* mRNA, consequently inhibiting the propagation of DNA virus, and increasing the yield of *Ifnb1* mRNA induced by dsDNA or HCMV. By comparison, knockout of ALKBH5 reduced the output of primary *Ifnb1* mRNA, but did not significantly affect the degradation rate of *Ifnb1* mRNA.^[^ ^26^ ^]^ It was shown that HMPV RNAs were m^6^A methylated and that m^6^A methylation restrained the binding efficiently of viral RNA to RIG‐I, which inhibited IFN expression and promoted HMPV replication.^[^ ^27^ ^]^ It was also reported that m^6^A modification of viral transcripts inhibited viral RNA recognition by RIG‐I and regulated host innate immunity against hepatitis B and C viral infections by inducing.^[^ ^28^ ^]^

As mentioned above, m^6^A modification play important roles on IFNB1 production, the main mechanisms focused on m^6^A modification on IFNB1 mRNA or virus RNA directly. Whether the vital participants in the RLR‐induced innate signaling pathway, like RIG‐1, MDA5, MAVS, TBK1, and IRF3, could be modified by RNA modification m^6^A is still unclear. It was reported that DDX46 RNA helicase interacted with m^6^A “eraser” ALKBH5 directly but not the writers like METTL3 or METTL14, which then demethylate m^6^A‐modified *Mavs*, TNF receptor‐associated factor 3 (*Traf3)*, and TNF receptor associated factor 6 (*Traf6)* transcripts. The m^6^A demethylation inhibited the mRNA translocation of those adaptors from nucleus to cytoplasm.^[^ ^25^ ^]^ However, there were still some unsolved problems in the paper. For example, ALKBH5 could demethylase m^6^A modification of *Mavs*, *Traf3*, and *Traf6* mRNA, that means the demethylation of m^6^A lacks specificity, and the m^6^A modification sites or the sequences similarity on the adaptors mRNA were not clearly identified. Moreover, the demethylation of m^6^A modification on *Mavs*, *Traf3*, and *Traf6* mRNA by AKBH5 depended on the existence of DDX46; the role of m^6^A “writers” was not elaborated. Thereby, different “writers” might play distinct roles on those adaptors mRNA. Discovering new m^6^A targets and mechanisms in RLR‐induced innate signaling pathway is still a challenge in the field.

In this study, we found an evolutionarily conserved mechanism for regulating type I IFN response, in which METTL14 mediated m^6^A modification on *MAVS* mRNA specifically. Importantly, both knockdown and knockout of METTL14 could increase the stability and expression of *MAVS* mRNA, but not affect the nuclear translocation of *MAVS* mRNA, which is a different and new mechanism compared to previous reports. Using MeRIP‐seq and MeRIP‐PCR, we also confirmed that the *Mavs* mRNA was m^6^A‐modified by *Mettl14*. We used a luciferase reporter that included the wild‐type CDS truncation sequence (*Mavs*‐WT) and demonstrated that expressing wild‐type Flag‐METTL14 markedly weakened MAVS response, but not that of *Mavs*‐MUT, compared with empty vector. Thereby, we identified that the 131246518 site on *Mavs* mRNA was the modification targets of *Mettl14*. These data proved that *Mavs* mRNA was regulated by METTL14 through m^6^A modification and therefore affects its stability. Our study found an interesting phenomenon that m^6^A methylation of *Mavs* mRNA is decreased in virus‐induced macrophages (Figure 4B). We measured the expression of methyltransferases (METTL3, METTL14) and demethylases (FTO, ALKBH5), and found that the expression of FTO and ALKBH5 rather than METTL3 and METTL14 were gradually increased upon virus infection (Figure S4B, Supporting Information), which may account for that the m^6^A methylation of *Mavs* mRNA is decreased in virus‐induced macrophages (Figure 4B).

**Figure 7:**
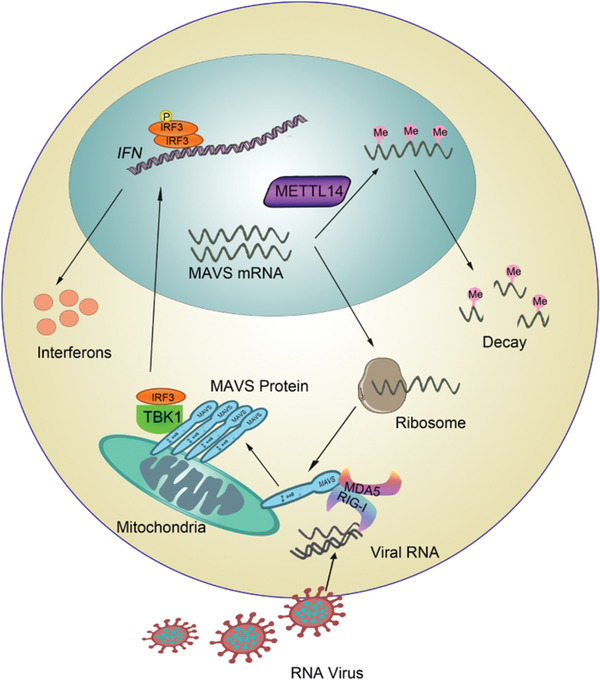
The proposed schematic model of METTL14 in RLR‐mediated innate immunity.

In conclusion, we found that METTL14 plays an important role in the RLRs‐mediated phosphorylation of IRF3, as well as transcription of IFNs and inflammatory cytokines. The m^6^A‐modified 131, 246, and 518 sites on *Mavs* mRNA by MTTL14 impaired its mRNA stability and antivirus innate immunology response. Our study suggested that m^6^A modification on *MAVS* transcripts by METTL14 negatively regulates the RLR‐induced innate immunity. Deficiency of METTL14 increased MAVS protein stability and IFN‐*β* production in response to RNA viruses (**Figure** **7**).

## 4 Experimental Section

### Cells and Viruses

HEK293T, THP‐1, and Hela cells were obtained from the American Type Culture Collection. The cells were cultured in DMEM (HyClone) supplemented with 10% FBS, 100 U mL^−1^ penicillin and 100 µg mL^−1^l streptomycin at 37 °C with 5% CO_2_. BMDMs were prepared as previously described.^[^ ^8^ ^]^ SeV was purchased from the China Center for Type Culture Collection (Wuhan University, China). VSV and VSV‐GFP were provided by H. Meng (Institute of Basic Medicine, Shandong Academy of Medical Sciences, China). The corresponding empty vectors were used as negative controls in all transfection experiments.

### Plasmids and Transfection

METTL14 cDNA was amplified from THP‐1 cells by standard PCR and cloned in pCMV2‐Flag plasmids. For dual‐luciferase reporter assay, the DNA fragments of wild‐type MAVS (*Mavs*‐WT) CDS truncation sequence (from 131245450 to 131246741) was amplified from macrophage and inserted downstream a firefly luciferase gene in pMIR‐REPORT vector (Luciferase miRNA Expression Reporter Vector, Ambion). Mutant plasmids for METTL14 R298P or *Mavs*‐MUT with an analogous sequence in which its putative m^6^A site was abolished by A to T mutations at 131246518 were made using the KOD‐Plus‐Mutagenesis kit (Toyobo, Osaka, Japan). Phorbol 12‐myristate 13‐acetate (PMA) was purchased from Invitrogen. Actinomycin‐D was from MedChemExpress. For transfection of plasmids into HEK293 cells, lipofectamine 3000 reagents were used (Invitrogen). For transfection of siRNA knockdown, THP‐1, BMDMs, or primary peritoneal macrophages were cultured in 24‐ or 6‐well plates, and siRNA transfection was performed using RNAIMAX (Invitrogen) by following the manufacturer's instruction. siRNAs were transfected into cells in each well. 48 h after transfection, knockdown efficiency was monitored by qPCR, or incubation continued to the subsequent indicated treatment. Small interfering sequences targeting METTL14 were as follows: mouse: 5′‐GCACCTCGGTCATTTATAT‐3′; human: 5′‐GGACUUCAUUCAUGCUAAUTT‐3′.

### Antibodies and Reagents

Antibodies used in this study were summarized at the dilutions listed: anti‐METTL14, 1:1000, (IB; Cell Signaling Technology, #: 51104S), anti‐N^6^‐methyladenosine modifications of RNA and DNA (m^6^A), 1:2000, (dot blot; Synaptic Systems, #: 202 003); anti‐IRF3, 1:1000, (IB; Cell Signaling Technology, #: 4302S); anti‐pIRF3, 1:1000, (IB; Cell Signaling Technology, #: 4947S) anti‐TBK1, 1:1000, (IB; Cell Signaling Technology, #: 3504S); anti‐pTBK1, 1:1000, (IB; Cell Signaling Technology, #: 5483S); anti‐RIG‐I, 1:1000, (IB; Cell Signaling Technology, #: D14G6); anti‐MDA5, 1:1000, (IB; Cell Signaling Technology, #: D74E4); anti‐FTO, 1:1000 (IB; Cell Signaling Technology, #: D6Z8W); anti‐METTL3, 1:1000 (IB; Cell Signaling Technology, #: E3F2A); anti‐ALKBH5, 1:1000 (IB; Abcam, #: ab195377); anti‐MAVS, 1:500, (IB; Santa Cruz Biotechnology, #: sc‐365333); anti‐FLAG (M2), 1:1000, (IB; Sigma‐Aldrich, #: F1804); anti‐*β*‐actin, 1:2000, (IB; ZSGB‐BIO, #:TA‐09). CHX (HY‐12320), actinomycin‐D, (HY‐17559), were obtained from MedChemExpress (MCE, NJ, USA); PMA (P1585) and Dynabeads mRNA Purification Kit (#: 61006) were purchased from Invitrogen; Click‐iT nascent RNA capture kit (#: C10365) was purchased from Life Technologies; EpiMark N6‐Methyladenosine Enrichment Kit (E1610S) was purchased from New England Biolabs.

### Animal Experiments

All animal experiments were carried out in agreement with the regulations of the National Institute of Health Guide for the Care and Use of Laboratory Animals, and approved by the Ethics Committee on Scientific Research of Shandong University Qilu Hospital, Jinan, Shandong Province, China. Permission‐numbers: KYLL‐2017(KS)‐361. All mice were housed in individually ventilated cages under specific pathogen‐free conditions. METTL14‐deficient mice were generated by Cyagen Biosciences Inc. (Guangzhou, China) using CRISPR/Cas system. Briefly, Cas9/sgRNA expression plasmid and targeting vector inserted with the upstream and downstream of the exon 7–10 of METTL14 were constructed. Linearized Cas9/sgRNA and targeting vector were micro‐injected into mouse zygote. Injected zygotes were implanted into C57BL/6 female mice. Genotyping was performed by PCR using the following primers: F1: 5′‐GCCTTCAGAGATGACGATGACTTC‐3′; F2: 5′‐CTGCCTAAAAGTCCTCCCTACTC‐3′; R1: 5′‐GTTACAAGAGGCCAGGTAAGAGTG‐3′.

All mice used were 6–8 weeks old. The mouse experiments were carried out following the general guidelines published by the Association for Assessment and Accreditation of Laboratory Animal Care. All of the mice were on the C57BL/6 background and were fed under specific‐pathogen free conditions with the approval of the Scientific Investigation Board of the Medical School of Shandong University.

### Viral Infection and Plaque Assay

HEK293 (2 × 10^5^), THP‐1 (5 × 10^5^), BMDMs (5 × 10^5^), or primary peritoneal macrophages (5 × 10^5^) were plated 24 h before infection. VSV (MOI, 0.1) or SeV were transduced into cell for the indicated times. VSV plaque assay and VSV replication were performed in HEK293 cells as previously described.^[^ ^29^ ^]^

### mRNA Isolation and Quantitative Reverse Transcription PCR

RNA was extracted from whole cell lysates using EASYspin Plus tissue/cell rapid RNA exaction kit (Aidlab) and 0.5 µg of total RNA was reverse transcribed with a PrimeScript RT reagent Kit (Takara). qPCR analysis was performed in triplicate wells with an iCycler IQ thermal cycler and detection system (Bio‐Rad) using the SYBR RT‐PCR kit (Roche) according to the manufacturer's instructions. The data were normalized to the expression of the actin housekeeping gene in each individual sample. The 2^−∆∆Ct^ method was utilized to calculate relative expression variations. Specific primers used for RT‐PCR assays are list as Table S1, Supporting Information.

### Virus Infection In Vivo

Littermate mice of *Mettl14* ^+/+^ and *Mettl14* ^+/−^ mice were allocated into groups according to age and sex and intraperitoneally (i.p.) injected with VSV (1 × 10^8^ PFU per mouse). Mice were sacrificed and serum IFN‐beta levels were measured using ELISA. The VSV titers in the spleen, lung, or liver were detected by standard plaque assays. Lungs from mice were dissected, fixed in 10% phosphate‐buffered formalin, embedded into paraffin, sectioned, stained with hematoxylin–eosin solution, and inspected by light microscopy for histological changes. For survival experiments, mice were evaluated for survival after VSV infection. To deplete the macrophages in vivo, 200 vL of clodronate liposome (FormuMax Scientific Inc.) or 200 vL of control liposome suspension (FormuMax Scientific Inc.) was intravenously administered to mice for 3 days and i.p. injected with VSV (5 × 10^7^ PFU per mouse) and then the next experiments were performed.

### Lentivirus Production and Infection

METTL14 and METTL14 (R298P) were cloned into the lentiviral expression vector pLVX‐IRES‐Puro. The viral particles were produced by transfecting HEK293T cells with METTL14‐ or METTL14 (R298P)‐expressing, or control plasmids in combination with pLVX‐IRES‐Puro, pMD2.G, and psPAX2 using Lipofectamine 3000 (Thermo Fisher Scientific). Viral supernatant was collected after 48 h. Macrophages were infected with lentivirus for at least 3 days and then the efficiency of expression was assessed by western blot 24 h after infection.

### Analysis of Nascent RNA Synthesis

To detect the transcription of newly born RNA, a Click‐iT nascent RNA capture kit (Life Technologies) was used. *Mettl14* ^+/+^ and *Mettl14* ^+/−^ peritoneal macrophages were exposed to a 30‐min EU pulse after SeV infection at the indicated times. Following EU exposure, cells were washed with PBS and were harvested, and RNA samples were extracted for qPCR analysis.

### RNA Decay Assays

Cells were seeded in 12‐well plates and cultured overnight at 37 °C. The next day, the authors performed the corresponding transfection operation. Cells were then infected for 8 h with SeV, followed by treatment with actinomycin‐D at a final concentration of 5 µg mL^−1^ for the indicated times. Cells were harvested and RNA samples were extracted for qPCR analysis to detect *MAVS* mRNA levels. The data were normalized to the *t* = 0 time point.

### m^6^A Dot Blot Assays

Total RNA was isolated from different cells according to the manufacturer's instructions. The m^6^A dot blot assay was executed following a published protocol^[^ ^30^ ^]^ with appropriate modifications. In brief, diluted RNA was heated at 95 °C for 3 min to disrupt secondary structure and then cooled down. The cold RNA samples were loaded on Amersham Hybond‐N^+^ membrane (GE Healthcare), dried, and fixed by UV cross‐linking. The membrane was blocked with 5% nonfat dry milk (in 1× PBST) for 1–2 h and incubated with a specific anti‐m^6^A antibody (1:2000 dilution, Synaptic Systems, 202003) overnight at 4 °C. After washing, the membrane was incubated with HRP‐conjugated goat anti‐rabbit IgG (1: 5000 dilution) for 1 h at room temperature. Blots were exposed with Pierce ECL Western Blotting Substrate (Thermo Fisher Scientific).

### Luciferase Reporter Assay

For dual‐luciferase reporter assay, HEK‐293T cells (0.5 × 10^5^) were seeded in 24‐well plate overnight and 200 ng *Mavs*‐WT or *Mavs*‐MUT, 200 ng Flag‐METTL14‐WT or Flag‐METTL14‐R298P plasmid, and 20 ng pRL‐TK (Renilla luciferase plasmid) were cotransfected for 48 h, followed by SeV infection for 8 h. The relative luciferase activities were determined using Dual‐Luciferase Reporter Assay System (Promega).

### m^6^A RNA Immunoprecipitation

Over 100 µg total RNA from *Mettl14* KO and WT peritoneal macrophages were extracted with TRIzol reagent (Invitrogen) and subjected to Poly(A)^+^ mRNA purification via Dynabeads mRNA Purification Kit (Invitrogen) according to the manufacturer's instructions. For m^6^A RNA, immunoprecipitation was performed using an EpiMark N^6^‐Methyladenosine Enrichment Kit (NEB). Briefly, 25 µL of Protein G Magnetic Beads (NEB #S1430) were washed twice with 200 µL reaction buffer (150 mm NaCl, 10 mm Tris‐HCl, pH 7.5, 0.1% NP‐40 in nuclease free H_2_O), and resuspended completely in 250 µL reaction buffer. 1 µL N^6^‐methyladenosine antibody or IgG antibody was added to the beads and incubated with orbital rotation for 30 min at 4 °C. Beads were washed twice with Reaction Buffer and resuspended in 250 µL reaction buffer. Poly(A) selected purified polyadenylated RNA was added to the resuspended beads and incubated with orbital rotation for 1 h at 4 °C. Beads were washed twice with Reaction Buffer, twice with Low Salt Reaction Buffer (50 mm NaCl, 10 mm Tris‐HCl, pH 7.5, 0.1% NP‐40 in nuclease free H_2_O), twice with High Salt Reaction Buffer (500 mm NaCl, 10 mm Tris‐HCl, pH 7.5, 0.1% NP‐40 in nuclease free H_2_O), and resuspended completely in 30 µL of Buffer RLT (Qiagen, 20 µL of Dynabeads MyOne Silane (Life Technologies,) was washed with 100 µL of Buffer RLT, resuspended in 30 µL of Buffer RLT, and added to the eluted RNA. Subsequently, 60 µL of 100% ethanol was added to the RNA and Dynabeads mixture. RNA‐bound beads were washed twice with 200 µL 70% ethanol and incubated with 16 µL nuclease‐free water for 1 min at room temperature to elute the RNA, followed immediately by cDNA synthesis.

### Statistical Analysis

For statistical analysis, GraphPad Prism 7.0 was used for all analyses. Unpaired Student's *t*‐test was applied to compare the differences between the two groups. All data were presented as mean ± standard deviation (SD) of one representative experiment. For the mouse survival study, Kaplan–Meier survival curves were generated and analyzed by the log‐rank Mantel–Cox test; All statistical tests were two‐tailed; *P* < 0.05 was considered statistically significant, *P < 0.05, **P < 0.01, NS: no significance

## Conflict of Interest

The authors declare no conflict of interest.

## Author Contributions

C.G. conceived and designed research; B.L. and F.Q. performed research; L.Z. and Y.Z. provided discussions; B.L., F.Q., and C.G. analyzed data; C.G. and B.L. wrote the paper.

## Supporting information

Supporting Information

## Data Availability

Research data are not shared.
