# Supplementary material for: Methyltransferase‐Like Protein 14 Attenuates Mitochondrial Antiviral Signaling Protein Expression to Negatively Regulate Antiviral Immunity via N6‐methyladenosine Modification
Source: Adv Sci (Weinh). 2021 May 27;8(15):2100606. doi: 10.1002/advs.202100606 (PMC8336497; doi:10.1002/advs.202100606)
Supplement: Supplementary file 1 — Supporting Information [file ADVS-8-2100606-s001.pdf]

© 2021 Wiley-VCH GmbH

ADVANCED SCIENCE
Open Access

Supporting Information

for *Adv. Sci.,* DOI: 10.1002/advs.202100606

METTL14 attenuates MAVS expression to negatively regulate antiviral immunity via m6A modification

Fei Qin[1], Baoshan Cai[1], Jian Zhao[1], Lei Zhang[1], Yi Zheng[1], Bingyu Liu[1,], Chengjiang Gao[1,2,*]*

Supporting Information

METTL14 attenuates MAVS expression to negatively regulate antiviral immunity via m^{6}A modification

Fei Qin[1], Baoshan Cai[1], Jian Zhao[1], Lei Zhang[1], Yi Zheng[1], Bingyu Liu[1,*], Chengjiang Gao[1,2,*]

[1]Key Laboratory of Infection and Immunity of Shandong Province & Department of Immunology, School of Biomedical Sciences, Shandong University, Jinan, Shandong 250012, P. R. China

[2]Lead Contact

[*]Correspondence: Dr. Chengjiang Gao

E-mail: cgao@sdu.edu.cn

Or Dr. Bingyu Liu

E-mail: liubingyu@sdu.edu.cn

Figure S1. Knockdown METTL14 inhibits RLR-induced innate immunity signaling.

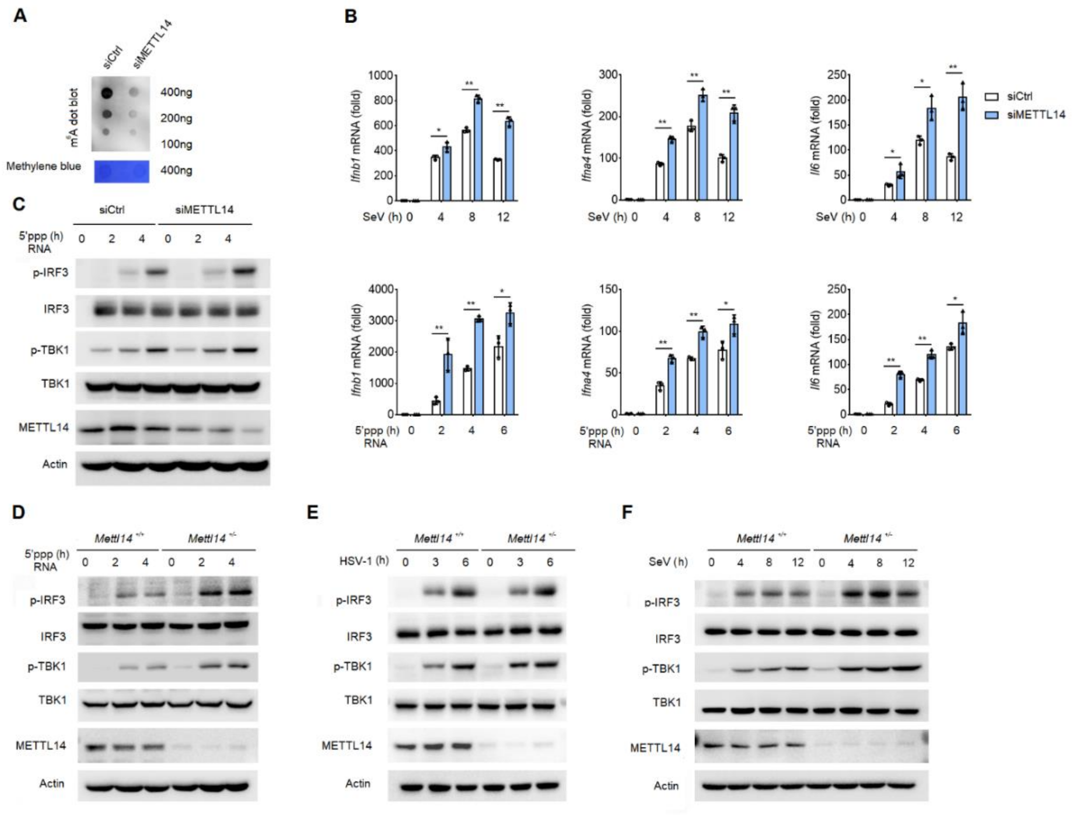

A). m^{6}A dot blot assays of peritoneal macrophages transfected with control siRNA (siCtrl) or siRNA targeting METTL14 (siMETTL14) for 48 h; Methylene blue staining (as loading control).

B). qPCR analysis of *Ifnb1*, *Il6* or *Ifna4* mRNA expression in primary macrophages transfected with that siRNA for 48 h, followed by infection with SeV or transfection with 5′-ppp RNA for the indicated times.

C). Immunoblot analysis of phosphorylated and total IRF3 and TBK1 in lysates of peritoneal macrophages transfected with control siRNA (siCtrl) or siRNA targeting METTL14 (siMETTL14) for 48 h, followed by transfection with 5′-pppRNA for the indicated times.

D). Immunoblot analysis of phosphorylated and total IRF3 and TBK1 in lysates of *Mettl14*$^{+/+}$ and *Mettl14*$^{+/-}$ mice macrophages transfected with 5′-pppRNA for the indicated times.

E). Immunoblot analysis of phosphorylated and total IRF3 and TBK1 in lysates of *Mettl14*$^{+/+}$ and *Mettl14*$^{+/-}$ mice macrophages infected with HSV-1 for the indicated times.

F). Immunoblot analysis of phosphorylated and total IRF3 and TBK1 in lysates of *Mettl14*$^{+/+}$ and *Mettl14*$^{+/-}$ mice BMDMs infected with SeV for the indicated times.

Data information: Data are presented as mean ± S.D. (B) Two-tailed unpaired Student's t-test; *$P < 0.05$; **$P < 0.01$.

Figure S2. METTL14 attenuates MAVS protein expression in BMDMs and peritoneal macrophages.

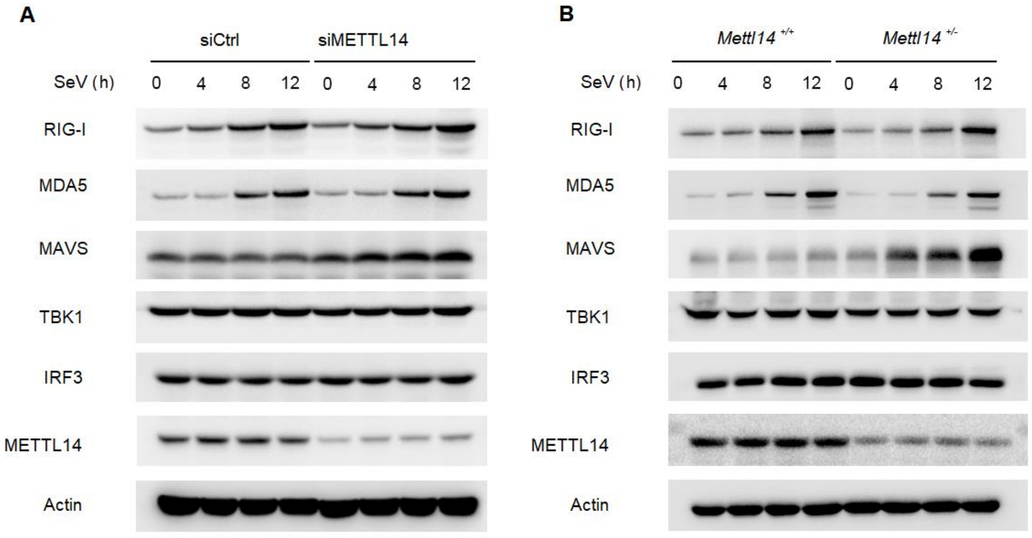

A). Immunoblot analysis of the main adaptors in RLRs signaling pathway in peritoneal macophages transfected with control siRNA (siCtrl) or siRNA targeting METTL14 (siMETTL14) for 48 h, followed by infection with SeV for the indicated times.

B). Immunoblot analysis of the main adaptors in RLRs signaling pathway in *Mettl14*$^{+/+}$ and *Mettl14*$^{+/-}$ BMDMs infected with SeV for the indicated times.

Figure S3. METTL14 promotes MAVS mRNA decay

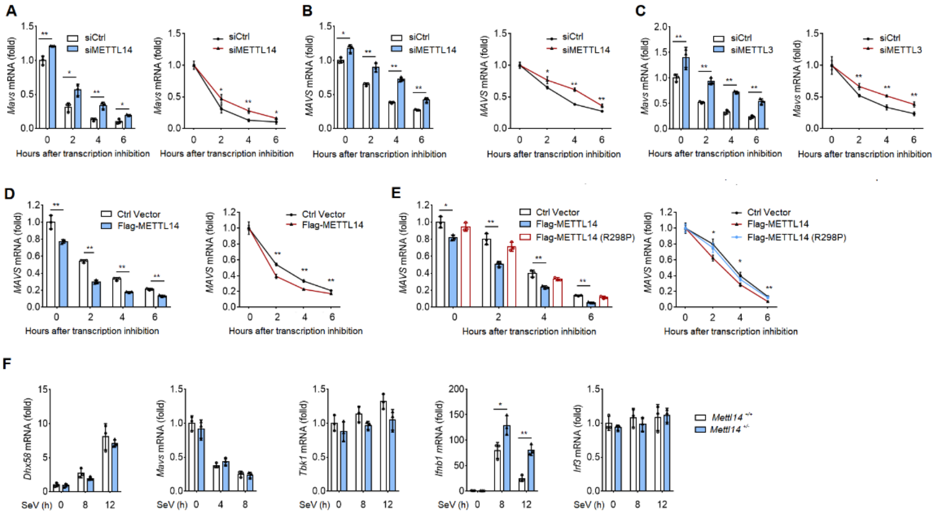

A) . qPCR analysis of *Mavs* mRNAs (left) and *Mavs* mRNA degradation (right) in peritoneal macrophages transfected with control siRNA (siCtrl) or siRNA targeting METTL14 (siMETTL14) for 48 h, followed by infection with SeV for 8 h and treatment with actinomycin D as indicated times.

B). qPCR analysis of *Mavs* mRNAs (left) and *Mavs* mRNA degradation (right) in THP-1 cells transfected with control siRNA (siCtrl) or siRNA targeting METTL14 (siMETTL14) for 48 h, followed by infection with SeV for 8 h and treatment with actinomycin D as indicated times.

C). qPCR analysis of *Mavs* mRNAs (left) and *Mavs* mRNA degradation (right) in peritoneal macrophages transfected with control siRNA (siCtrl) or siRNA targeting METTL3 (siMETTL3) for 48 h, followed by infection with SeV for 8 h and treatment with actinomycin D as indicated times.

D). qPCR analysis of *Mavs* mRNAs (left) and *Mavs* mRNA degradation (right) in HEK293T cells transfected with control plasmid or plasmid expressing Flag-METTL14 for 16 h, followed by infection with SeV for 8 h and treatment with actinomycin-D as indicated.

E). qPCR analysis of *Mavs* mRNAs (left) and *Mavs* mRNA degradation (right) in HEK293T cells transfected with Flag-METTL14-WT or Flag-METTL14-R298P plasmid for 16 h, followed by infection with SeV for 8 h and treatment with

actinomycin-D as indicated times.

F). qPCR analysis of *Ddx58*, *Mavs*, *Tbk1*, *Ifnb1*, *Irf3* mRNAs in *Mettl14*$^{+/+}$ and *Mettl14*$^{+/-}$ peritoneal macrophages were exposed to a 30-min EU pulse after SeV infection for the indicated times.

Data information: Data are presented as mean ± S.D. (A-F) Two-tailed unpaired Student's t-test; **P* < 0.05; ***P* < 0.01.

Figure S4. The m^{6}A modification of the main adaptors in RLRs signaling pathway catalyzed by METTL14

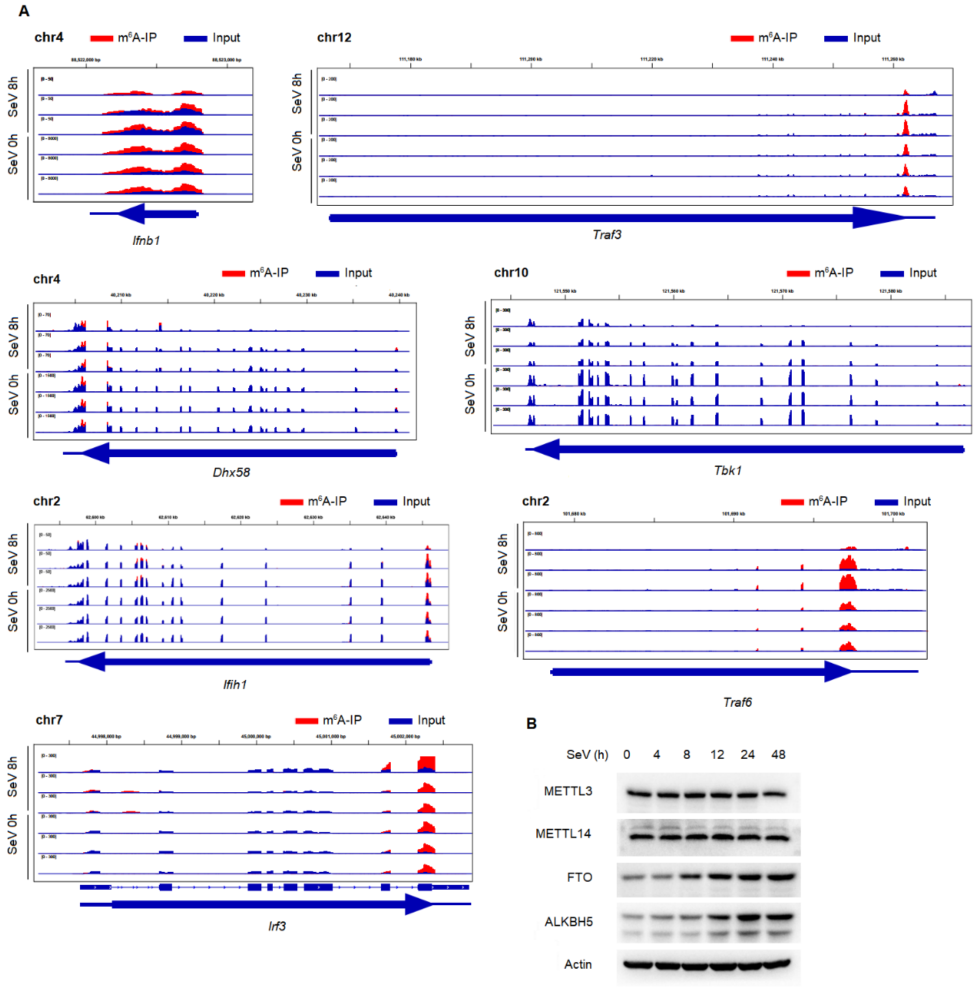

A). RNA-seq of *Ifnb1*, *Ddx58*, *Ifih1*, *Tbk1*, *Irf3*, *Traf3*, *Traf6* mRNAs in input RNA and m^{6}A immunoprecipitated RNA from peritoneal macrophages infected with SeV for 8h.

B). Immunoblot analysis of m^{6}A machinery proteins upon SeV infection in primary macrophages.

Figure S5. Knockdown of METTL14 inhibits cellular antiviral response to RNA virus

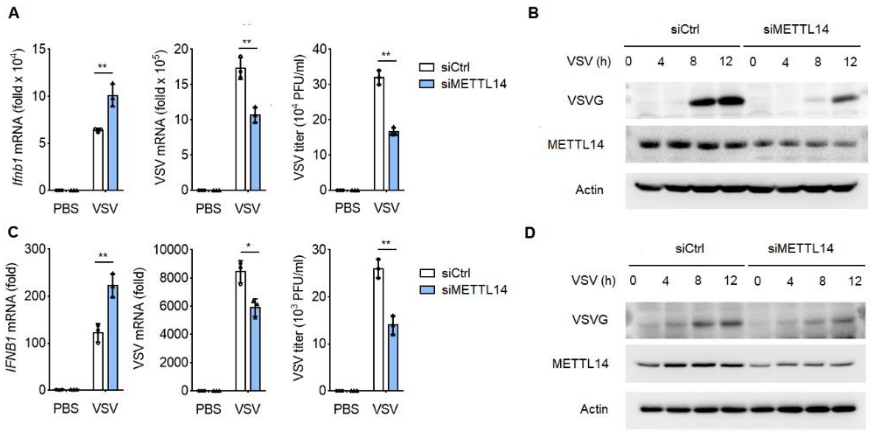

A). qPCR analysis of *Ifnb1* mRNA (left), VSV mRNA (middle) and plaque assay of VSV titers (right) in primary macrophages transfected with METTL14 siRNA for 48 h, followed by infected with VSV (MOI, 0.1) for 12 h; PFU, plaque-forming units.

B). Immunoblot analysis of VSV-G in mouse primary peritoneal macrophages transfected with siRNA for 48 h as in (A), followed by infection with VSV (MOI, 0.1) for the indicated times.

C). qPCR analysis of *IFNB1* mRNA (left), VSV mRNA (middle) and plaque assay of VSV titers (right) in THP-1 cells transfected with control siRNA (siCtrl) or siRNA targeting METTL14 (siMETTL14) for 48 h, followed by infection with VSV (MOI, 0.1) for 12 h.

D). Immunoblot analysis of VSV-G in THP-1 cells transfected as in (C), followed by infection with VSV (MOI, 0.1) for the indicated times.

Data information: Data are presented as mean ± S.D. (A, C) Two-tailed unpaired Student’s t-test; *$P < 0.05$; **$P < 0.01$.

Figure S6. *Mettl14* facilitates VSV replication *in vivo* by macrophages.

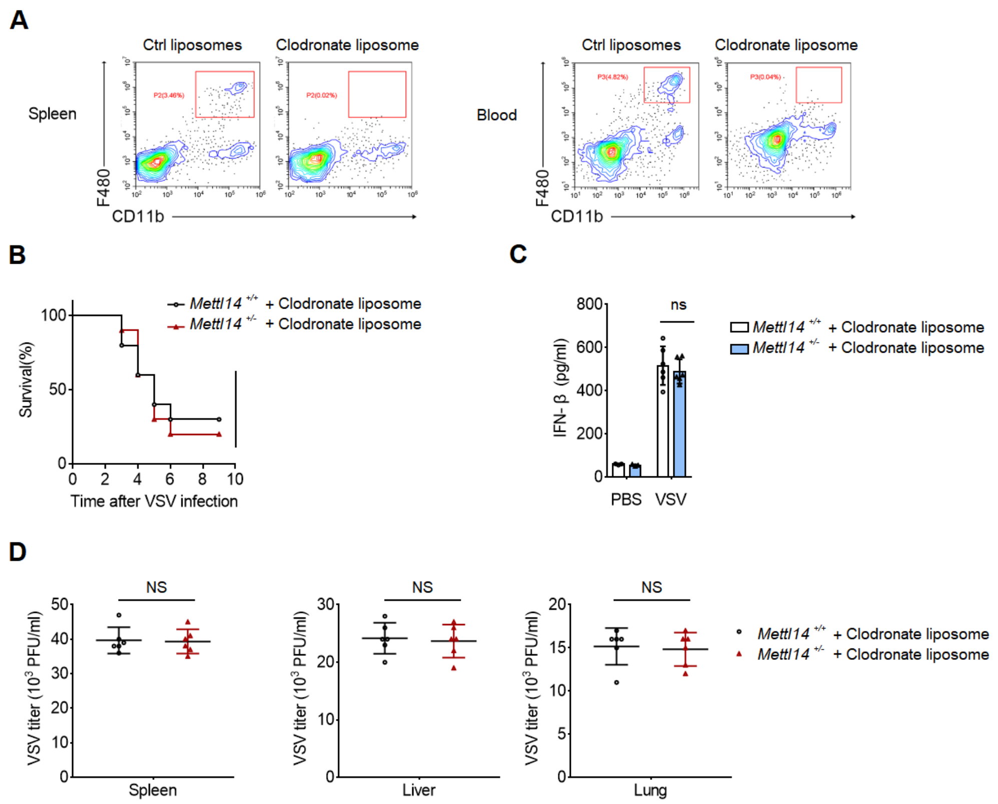

A). Representative flow cytometric analysis of spleen macrophages and blood macrophages of control liposome and clodronate liposome-treated mice on day-3 after

injection.

B). Survival of *Mettl14*$^{+/+}$ and *Mettl14*$^{+/-}$ mice treated with clodronate liposome and infected intravenously with VSV (5×10^7 PFU per mouse).

C). ELISA analysis of IFN-β of Serum from *Mettl14*$^{+/+}$ and *Mettl14*$^{+/-}$ mice treated with clodronate liposome and infected intraperitoneally with VSV (5×10^7 PFU per mouse) for 8h.

D). Plaque assay of VSV titers of the spleen, liver and lungs from *Mettl14*$^{+/+}$ and *Mettl14*$^{+/-}$ mice treated with clodronate liposome and infected by intraperitoneal injection of VSV (5×10^7 PFU per mouse) for 48 h. Each symbol represents an individual mouse; small horizontal lines indicate the mean.

Data information: Data are presented as mean ± S.D. (B) log-rank test; (C, D) Two-tailed unpaired Student's t-test; NS: no significance.

Table S1 Primers for RT-qPCR

Gene	Forward	Reverse
Mouse *Il*-6	ACAACCACGGCCTTCCCTAC	CATTTCCACGATTTCCCAGA
Mouse *Ifna4*	ACCCACAGCCCAGAGAGTGACC	AGGCCCTCTTGTTCCCGAGGT
Mouse *Ifnb*	AGATCAACCTCACCTACAGG	TCAGAAACACTGTCTGCTGG
Mouse *Mavs*	TGTGGACCTTGCCATTAG	GCATCACTGCCAGGAATA
Mouse *Irf3*	GAAAGAAGTGTTGCGGTTAG	GGCTTGGCAGTTGTTGAG
Mouse *Tbk1*	TCAGGCACTGCTTACCC	CGGCTCGTGACAAAGATAG
Mouse *Ddx58*	GGCGTTGGAGATGCTA	GCTGCTTCTCGGACAT
Human *IFNB1*	CAACAAGTGTCTCCTCCAAAT	TCTCCTCAGGGATGTCAAAG
Human *IL-6*	TGCAATAACCACCCCTGACC	TGCGCAGAATGAGATGAGTTG
Human *MAVS*	GAGCCACCGTCACTTCCT	GGGTACTGCCCAGTTCTGT
VSV	ACGGCGTACTTCCAGATGG	CTCGGTTCAAGATCCAGGT
